# Permanent relief from intermittent cold stress-induced fibromyalgia-like abnormal pain by repeated intrathecal administration of antidepressants

**DOI:** 10.1186/1744-8069-7-69

**Published:** 2011-09-21

**Authors:** Michiko Nishiyori, Hitoshi Uchida, Jun Nagai, Kohei Araki, Takehiro Mukae, Shiroh Kishioka, Hiroshi Ueda

**Affiliations:** 1 Division of Molecular Pharmacology and Neuroscience, Nagasaki University Graduate School of Biomedical Sciences, 1-14 Bunkyo-machi, Nagasaki 852-8521, Japan; 2 Department of Pharmacology, Wakayama Medical University, 811-1 Kimiidera, Wakayama 641-0012, Japan

**Keywords:** fibromyalgia, cold stress, vicious circle, antidepressant, allodynia, hyperalgesia

## Abstract

**Background:**

Fibromyalgia (FM) is characterized by chronic widespread pain, which is often refractory to conventional painkillers. Numerous clinical studies have demonstrated that antidepressants are effective in treating FM pain. We previously established a mouse model of FM-like pain, induced by intermittent cold stress (ICS).

**Results:**

In this study, we find that ICS exposure causes a transient increase in plasma corticosterone concentration, but not in anxiety or depression-like behaviors. A single intrathecal injection of an antidepressant, such as milnacipran, amitriptyline, mianserin or paroxetine, had an acute analgesic effect on ICS-induced thermal hyperalgesia at post-stress day 1 in a dose-dependent manner. In addition, repeated daily antidepressant treatments during post-stress days 1-5 gradually reversed the reduction in thermal pain threshold, and this recovery was maintained for at least 7 days after the final treatment. In addition, relief from mechanical allodynia, induced by ICS exposure, was also observed at day 9 after the cessation of antidepressant treatment. In contrast, the intravenous administration of these antidepressants at conventional doses failed to provide relief.

**Conclusions:**

These results suggest that the repetitive intrathecal administration of antidepressants permanently cures ICS-induced FM pain in mice.

## 2. Background

Fibromyalgia (FM) is characterized by generalized tenderness and chronic widespread pain that affects 2-4% of the population in industrialized nations and primarily affects females [1]. Although its etiology and pathogenesis are largely unknown, emerging evidence indicates that pain amplification within the central nervous system (CNS) plays a critical role in the pathology of FM pain [2]. Recent studies, including functional imaging, have revealed that this central amplification process depends, in part, on deficits in endogenous descending pain inhibitory pathways [3,4] and abnormal pain processing [5]. In addition, FM pain is often refractory to treatment using conventional painkillers, such as non-steroidal anti-inflammatory drugs and opioids [6]. However, numerous studies have demonstrated the effectiveness of antidepressants and antiepileptics, such as gabapentin and pregabalin, in the treatment of FM pain [7,8].

There are several animal models of FM pain, induced by either intramuscular injection of acidic saline [9], vagotomy [10], sound stress [11] or depletion of biogenic amines [12]. However, in order to better understand the molecular basis of the underlying pain mechanisms, it is necessary to establish an animal model which accurately reflects the pathological and pharmacotherapeutic features of the disease.

Recently, we established a mouse model of FM using intermittent cold stress (ICS), which produces long-lasting thermal hyperalgesia and mechanical allodynia, predominantly in females [13]. We found that gabapentin, particularly when injected intracerebroventricularly, had potent anti-hyperalgesic and anti-allodynic effects in this model [13]. In addition, systemically and intracerebroventricularly-administered morphine was found to have no analgesic effect in ICS-exposed mice, due to a failure to activate descending pain inhibitory pathways [14]. These findings indicate that our ICS model might accurately reflect the pathological and pharmacotherapeutic features of FM pain. In this study, we examine whether various antidepressants can ameliorate the abnormal pain sensations in this model.

## 3. Materials and methods

### 3.1. Animals

Male C57BL/6J mice weighing 18-22 g were used. They were kept in a room with an ambient temperature of 21 ± 2°C, with free access to a standard laboratory diet and tap water. All procedures were approved by the Nagasaki University Animal Care Committee and complied with the recommendations of the International Association for the Study of Pain [15].

### 3.2. Drug treatments

Antidepressants were obtained from Sigma (St. Louis, MO, USA). Milnacipran, paroxetine, and amitriptyline were dissolved in artificial cerebrospinal fluid (aCSF; 125 mM NaCl, 3.8 mM KCl, 2.0 mM CaCl_2_, 1.0 mM MgCl_2_, 1.2 mM KH_2_PO_4_, 26 mM NaHCO_3_, 10 mM glucose, pH 7.4). Mianserin was dissolved in physiological saline. For vehicle treatments, aCSF or saline was injected. Intrathecal (i.t.) injections were administered according to Hylden and Wilcox [16] using a 30-gauge needle. The site of injection was chosen to be between spinal L5 and L6--near where the spinal cord ends and the cauda equina begins. This allowed us to maximize inter-vertebral accessibility and to minimize the possibility of spinal damage. After sufficient training, the experimenters were able to perform the technique without causing injury to the animals.

### 3.3. Experimental model of fibromyalgia

**Figure 1:**
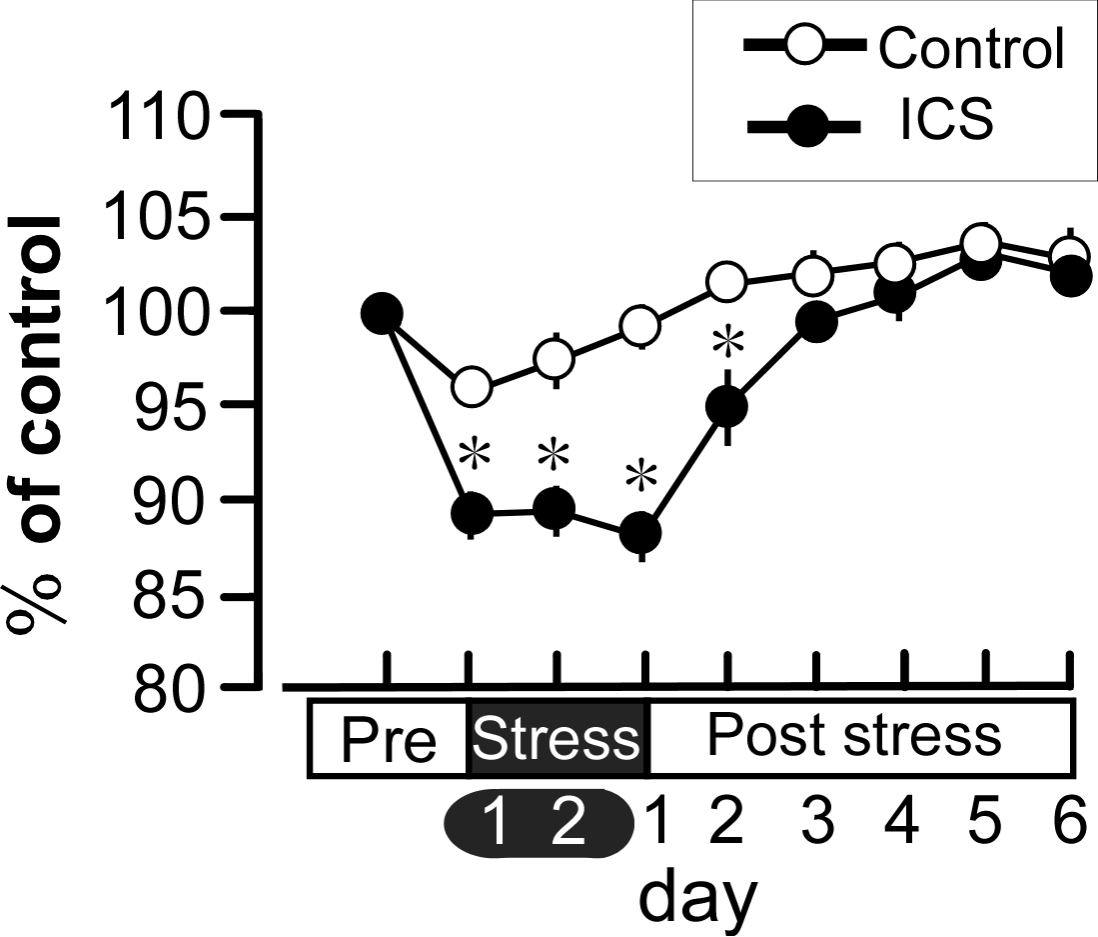
**Changes in body weight in ICS treated mice**. Results represent the percentage of body weight of mice, compared to the value at 1 day before ICS stress (Pre). *p < 0.05, *vs*. control group. Data are the means ± S.E.M; 6 mice per group.

ICS exposure and constant cold stress (CCS) were performed as previously reported [13]. Briefly, for the ICS model, mice were placed on stainless mesh plate in a cold room at 4°C overnight (from 4:30 pm to 10:00 am), followed by ICS with environmental temperatures alternating between 24 and 4°C every 30 min, from 10:00 am to 4:30 pm. These procedures were repeated twice. On day 3, the mice were adapted to 24°C for 1 h before behavior testing. We designated day 3 following the onset of stress exposure as day 1 post-stress exposure (P1). For the CCS model, mice were placed in the cold room from 4:30 pm on day 1 to 10:00 am on day 3, followed by adaptation at 24°C for 1 h. Mice in the control group were kept at 24°C for all 3 days (from 4:30 pm on day 1 to 10:00 am on day 3). During the stress period, two mice were kept in each cage (12 × 15 × 10.5 cm), with free access to food and agar as alternate drink water in place of fluid. Although the body weight of mice was decreased during and after the ICS stress, it attained to the control mice level as early as 4 day after the stress (Figure 1).

### 3.4. Measurement of plasma corticosterone

Plasma corticosterone levels were measured as described previously [17]. Briefly, plasma was separated by centrifugation at 3 000 *g *for 15 min at 4°C and collected into ice-chilled tubes containing 0.1% EDTA and stored at -80°C until use. Blood samples were collected at 9:00 pm in order to exclude the effect of circadian rhythms on circulating plasma corticosterone. The plasma corticosterone level was estimated fluorometrically, according to the method of Zenker and Bernstein [18].

### 3.5. Assessment of stress-related behaviors

Spontaneous locomotor activity was measured in the open filed (22 × 33 cm) for 3 min, using SCANET apparatus (Melquest, Japan). In the elevated plus-maze test used to estimate anxiety, the time spent in the open arm was recorded during a 6-min period. To assess depression-like behaviors, the tail-suspension test was performed [19,20]. Mice were suspended 30 cm above the floor using adhesive tape, and the total duration of immobility during a 6-min period was measured.

### 3.6. Nociception tests

In the thermal paw withdrawal test, the nociception threshold was assessed using the latency of paw withdrawal upon a thermal stimulus [21,22]. Unanesthetized animals were placed in plexiglass cage on top of a glass sheet and acclimated for 1 h. A thermal stimulator (IITC Inc., Woodland Hills, CA, USA) was positioned under the glass sheet and the focus of the projection bulb was aimed exactly at the middle of the plantar surface of the animal. A mirror attached to the stimulator permitted visualization of the plantar surface. A cut-off time of 20 s was set to prevent tissue damage.

The mechanical paw pressure test was performed as described previously [22]. Briefly, mice were placed in a plexiglass chamber on a 6 × 6 mm wire mesh grid floor and allowed to acclimate for a period of 1 h. A mechanical stimulus was then delivered to the middle of the plantar surface of the right-hind paw using a Transducer Indicator (Model 1601; IITC Inc., Woodland Hills, CA, USA). The pressure needed to induce a flexor response was defined as the pain threshold. A cut-off pressure of 20 g was set to avoid tissue damage. In these experiments using thermal and mechanical tests, the thresholds were determined from three repeated challenges at 10 min intervals, and the averages were used for statistical analysis. For the time-course experiments, we measured the paw-withdrawal latencies (PWL) at 30, 60, and 180 min after intrathecal injection of antidepressant. In the area under the curve (AUC) analysis of antidepressant-induced analgesia, we calculated the AUC generated by plotting analgesic threshold (after deducting the control threshold from each threshold point) against time, from 30 to 180 min after antidepressant treatment, using a trapezoidal method.

### 3.7. Statistical analysis

**Figure 2:**
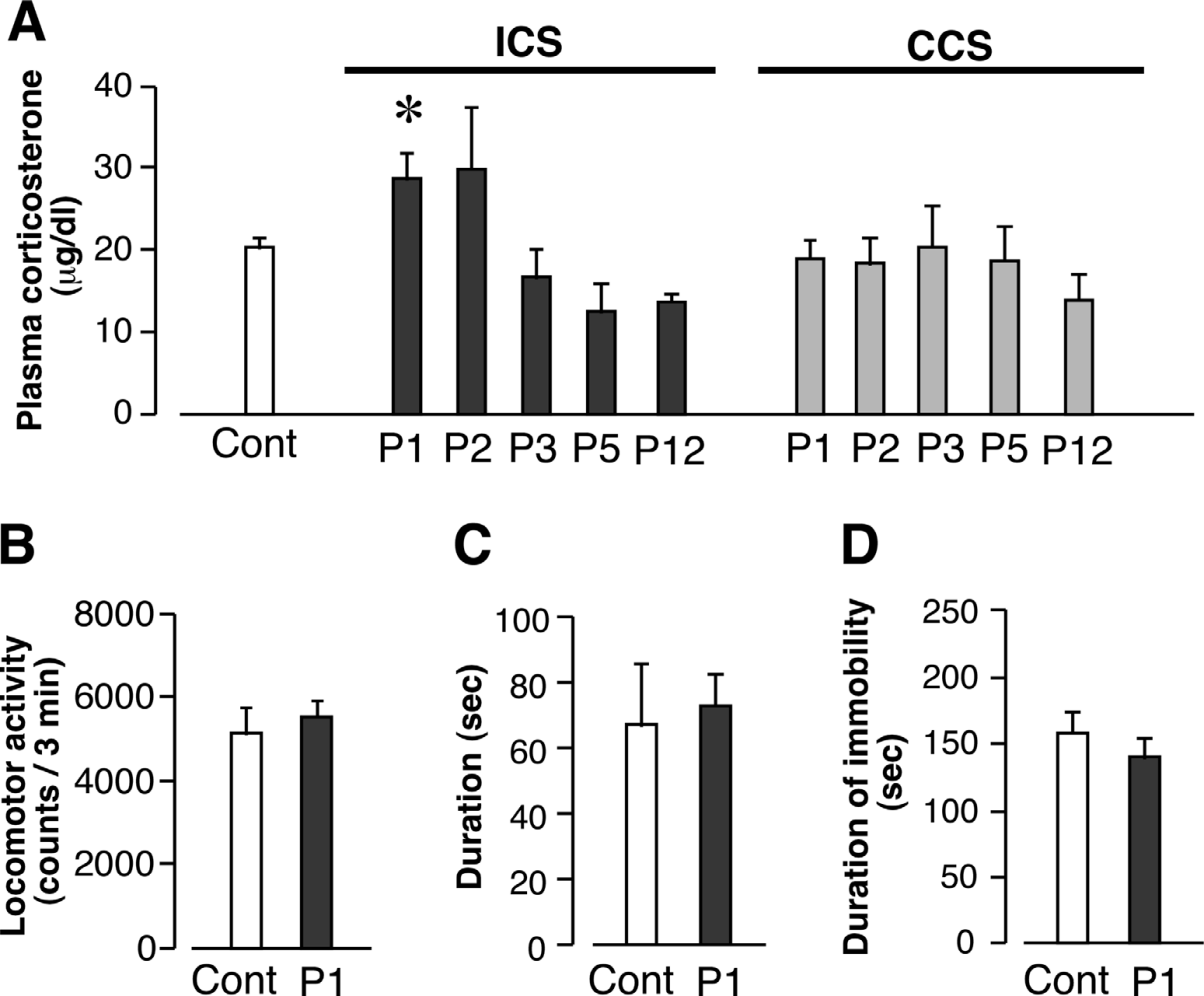
**Effects of stress exposure on plasma corticosterone levels, spontaneous locomotor activity, and anxiety and depression-like behaviors**. (A) Time-course of plasma corticosterone levels after ICS or CCS exposure. (B-D) Lack of changes in spontaneous locomotor activity in the SCANET apparatus (B), duration of time spent in the open arm of the plus-maze test (C), and total duration of immobility in the tail suspension test (D) at P1. *p < 0.05, *vs*. control group. Data are expressed as the means ± S.E.M; 3-5 mice per group.

**Figure 3:**
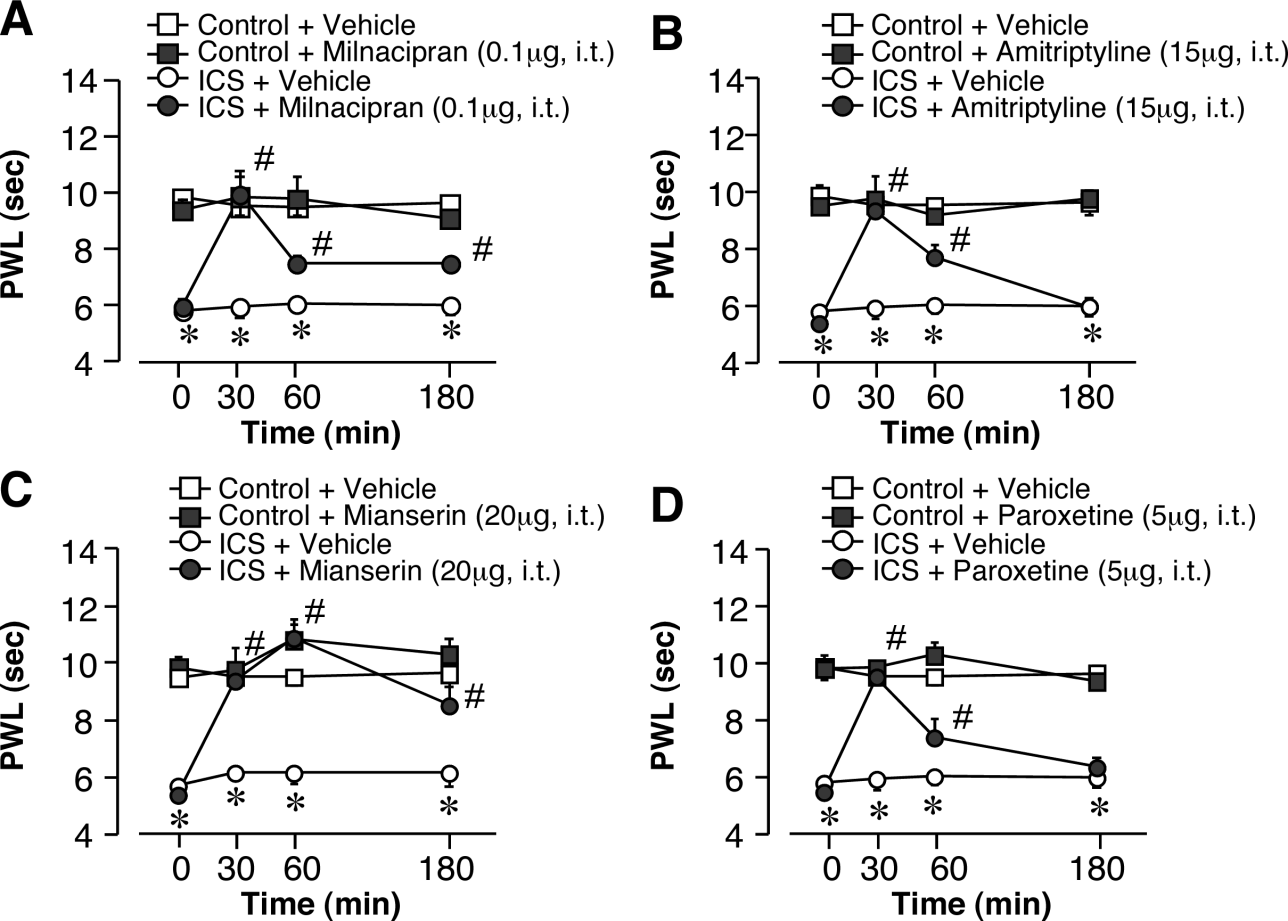
**Antidepressant-induced acute analgesic effects in ICS treated mice**. Thermal pain threshold was assessed at P1 after control or ICS treatment, using the thermal paw withdrawal test. Results represent the time course of thermal paw withdrawal latencies (PWL, in seconds) after a single intrathecal injection of antidepressants. (A-D) Each data point in [control + vehicle] and [ICS + vehicle] groups is common. *p < 0.05, *vs*. vehicle-treated control group; #*p *< 0.05, *vs*. vehicle-treated and ICS-exposed groups. Data are expressed as the means ± S.E.M.; 4-8 mice per group.

**Figure 4:**
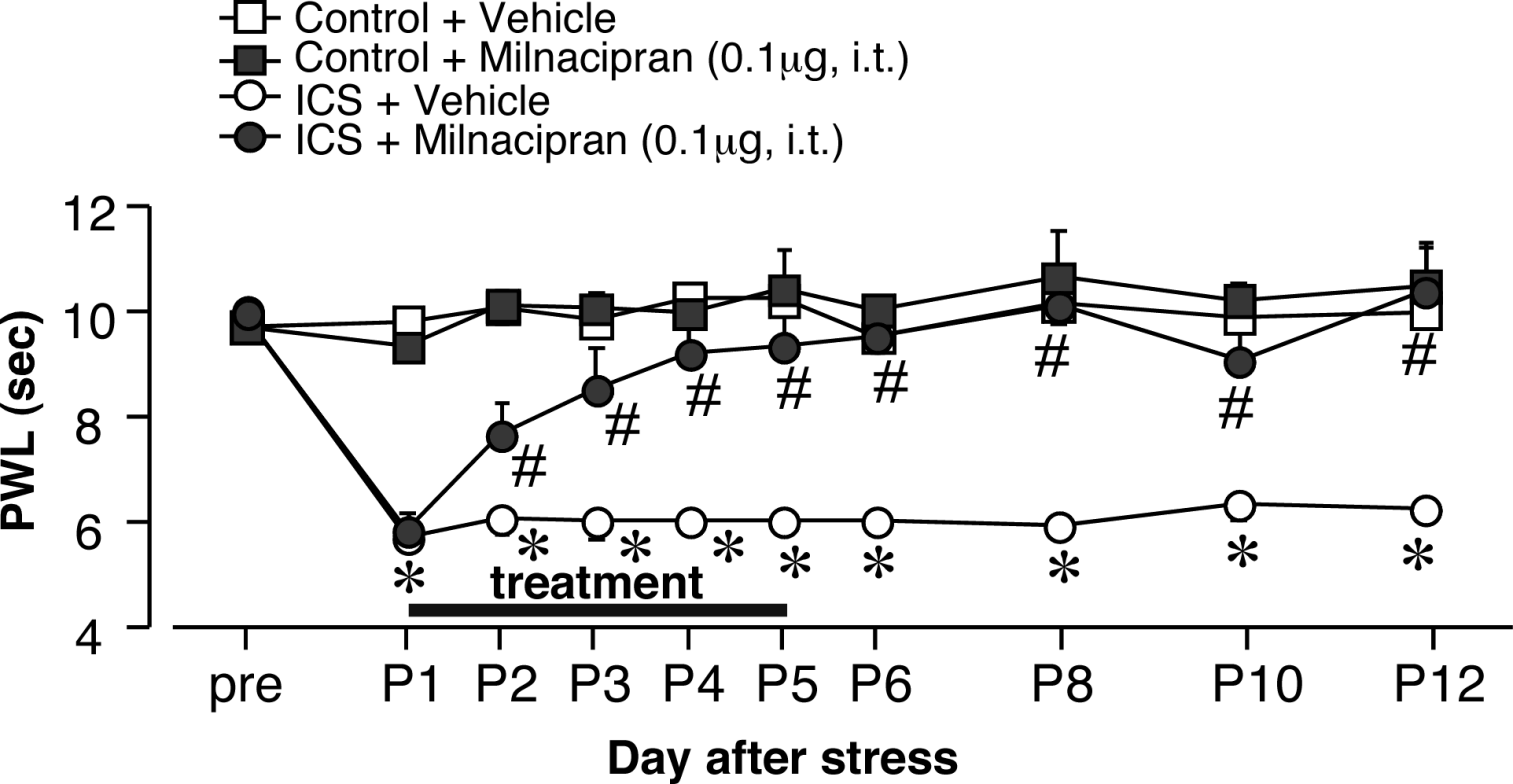
**Permanent relief from ICS-induced thermal hyperalgesia by repeated intrathecal administration of milnacipran**. Intrathecal injections of milnacipran (0.1 μg) were given once daily at 11:30 a.m. from P1-P5 after assessment of nociceptive thresholds at 11:00 a.m. Results represent the basal threshold as the latency to paw-withdrawal from thermal stimuli (PWL, in seconds), just before the daily injection of vehicle or milnacipran. *p < 0.05, *vs*. vehicle-treated control group; #*p *< 0.05, *vs*. vehicle-treated and ICS-exposed groups. Data are expressed as the means ± S.E.M.;4-8 mice per group.

**Figure 5:**
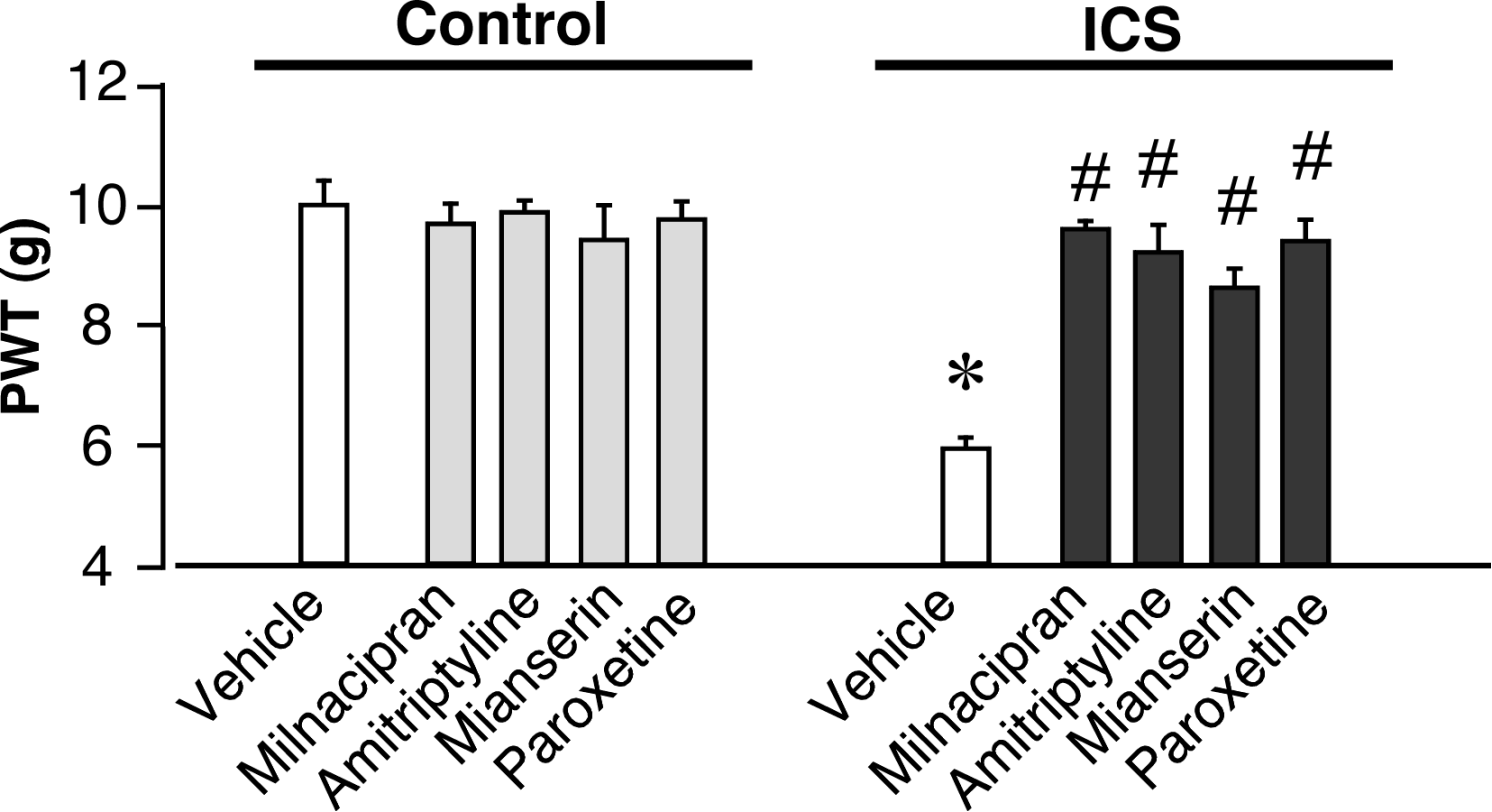
**Complete relief from ICS-induced mechanical allodynia**. Basal mechanical paw-withdrawal threshold (PWT, in grams) was assessed at P14, using paw pressure tests. Intrathecal injection of milnacipran (0.1 μg), amitriptyline (15 μg), mianserin (20 μg), or paroxetine (5 μg), was given once daily from P1-P5, as described in Figure 4. *p < 0.05, *vs*. vehicle-treated control group; #*p *< 0.05, *vs*. vehicle-treated and ICS-exposed groups. Data are expressed as the means ± S.E.M.; 3-6 mice per group.

**Figure 6:**
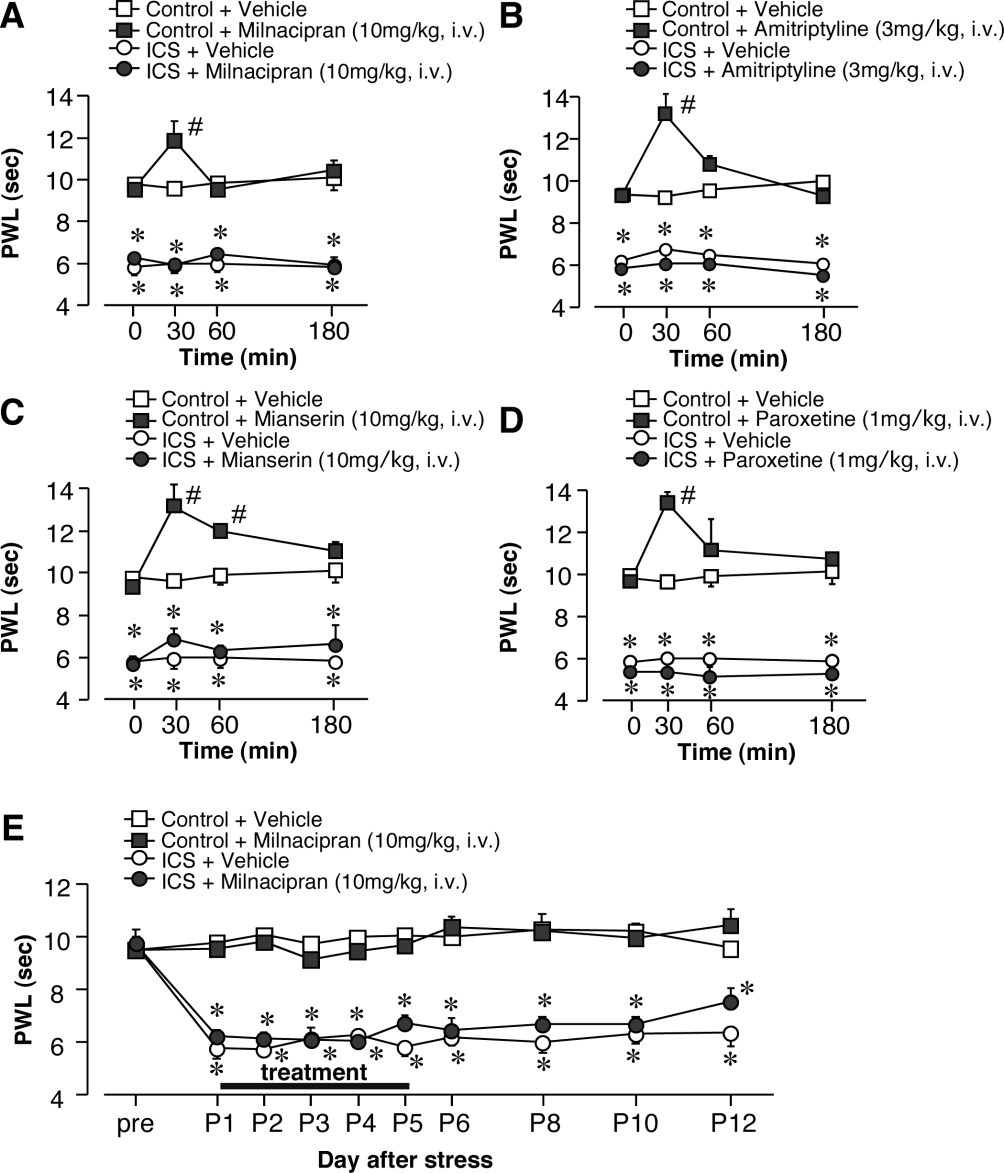
**Lack of anti-hyperalgesic effects by systemic administration of antidepressants**. Thermal pain threshold was assessed at P1 after control or ICS-treatment, using thermal paw withdrawal tests. (A-D) Results represent the time-course of thermal paw-withdrawal latencies (PWL, in seconds) after a single i.v. injection of antidepressants. (A-D) Each data point in [control + vehicle] and [ICS + vehicle] groups is common. (E) Milnacipran was given i.v. once daily for 5 days, as described in Figure 4. Results represent the basal threshold as the latency to paw-withdrawal from thermal stimuli (PWL, in seconds), just before the daily injection of vehicle or milnacipran. *p < 0.05, *vs*. vehicle-treated control group; #*p *< 0.05, *vs*. vehicle-treated and ICS-exposed groups. Data are expressed as the means ± S.E.M.; 3-6 mice per group.

In Figure 2 and Table 1, data were analyzed using Student's *t*-test. In Figures 1, 3, 4, 5 and 6, Tables 2 and 3, the differences between multiple groups were analyzed using a one-way ANOVA with the Tukey-Kramer multiple comparison post-hoc analysis. Significance was set at *p *< 0.05. All results are expressed as means ± S.E.M.

**Table 1:** Dose-dependent acute analgesic effects of antidepressants on ICS-induced thermal hyperalgesia

Drug	Dose (μg)	n	AUC
**Milnacipran**	0.03	4	50.6 ± 21.5

	0.1	6	281.9 ± 71.3*

	0.3	4	166.2 ± 31.3*

			

**Amitriptyline**	5	3	51.1 ± 85.2

	15	7	252.6 ± 42.2*

	30	3	235.5 ± 64.4*

			

**Mianserin**	10	3	144.7 ± 171.5

	20	4	527.8 ± 103.2*

			

**Paroxetine**	2	3	40.7 ± 62.4

	5	7	211.2 ± 38.6*

	10	3	251.3 ± 797*

Thermal pain threshold was assessed at P1, using thermal paw withdrawal tests. Acute anti-hyperalgesic effects of milnacipran, amitriptyline, mianserin, and paroxetine were evaluated by AUC, as described in Materials and Methods. *p < 0.05, *vs*. vehicle-treated control group.

**Table 2:** Permanent relief from ICS-induced thermal hyperalgesia by repeated intrathecal (i.t.) administration of amitriptyline, mianserin or paroxetine

	n	Pre (sec)	P1 (sec)	P5 (sec)	P12 (sec)
**Control-vehicle** **(i.t.)**	8	9.84 ± 0.33	9.81 ± 0.45	10.24 ± 0.23	9.99 ± 0.22

**ICS-vehicle** **(i.t.)**	8	10.22 ± 0.28	5.76 ± 0.15*	6.04 ± 0.16*	6.28 ± 0.21*

**ICS- amitriptyline** **(15 μg, i.t.)**	4	10.48 ± 0.23	5.35 ± 0.36	8.44 ± 0.16#	9.35 ± 0.66#

**ICS-mianserin** **(20 μg, i.t.)**	4	9.68 ± 0.30	5.35 ± 0.36	8.13 ± 0.25#	9.95 ± 0.66#

**ICS- paroxetine** **(5 μg, i.t.)**	4	9.82 ± 0.44	5.47 ± 0.13	9.08 ± 0.46#	9.52 ± 0.21#

Antidepressants were administered between P1-P5, as described in Figure 3. Thermal pain threshold was assessed using the thermal paw withdrawal test. *p < 0.05, *vs*. vehicle-treated control group; #*p *< 0.05, *vs*. vehicle-treated and ICS-exposed groups.

**Table 3:** Lack of anti-hyperalgesic effects by repeated systemic administration of antidepressants

	n	Pre (sec)	P1 (sec)	P5 (sec)	P12 (sec)
**Control-vehicle** **(i.v.)**	8	9.51 ± 0.22	9.80 ± 0.18	10.00 ± 0.29	9.57 ± 0.30

**ICS-vehicle** **(i.v.)**	8	9.67 ± 0.23	5.78 ± 0.36	6.02 ± 0.12	6.36 ± 0.21

**ICS- amitriptyline** **(3 mg/kg, i.v.)**	4	9.55 ± 0.42	5.85 ± 0.37	5.79 ± 0.36	6.72 ± 0.35

**ICS-mianserin** **(10 mg/kg, i.v.)**	4	9.77 ± 0.34	5.14 ± 0.56	5.98 ± 0.39	6.66 ± 0.41

**ICS- paroxetine** **(1 mg/kg, i.v.)**	4	9.34 ± 0.36	5.29 ± 0.34	6.89 ± 0.30	6.96 ± 0.65

**ICS- milnacipran** **(10 mg/kg, i.v.)**	4	9.75 ± 0.47	6.25 ± 0.25	6.71 ± 0.21	7.54 ± 0.53

Amitriptyline, mianserin, paroxetine or milnacipran were given intravenously (i.v.) and assessment of basal nociceptive thresholds was performed as described in Figure 5. Thermal pain threshold was assessed using the thermal paw withdrawal test. *p < 0.05, *vs*. vehicle-treated control group.

## 4. Results

### 4.1. Effects of ICS stress exposure on plasma corticosterone levels and anxiety and depression-like behaviors

We previously designed an improved mouse model for dysautonomia, also referred to as the specific alternation of rhythm in temperature (SART) model [23], and found that ICS, but not CCS, caused long-lasting abnormal pain sensations [13]. In the present study, we used plasma corticosterone levels as a biomarker for stress. As shown in Figure 2A, we found that ICS exposure caused a transient increase in plasma corticosterone levels at P1. In contrast, CCS exposure had no effect on plasma corticosterone levels between P1 and P12 (Figure 2A). ICS had no effect on spontaneous locomotor activity at P1 (Figure 2B). Furthermore there was no significant change in the duration of time spent in the open arm in the elevated plus-maze test or in the total duration of immobility in the tail-suspension test at P1 (Figures 2C, D). In addition, there were no gross behavioral changes in mice as early as 1 h after the transfer from 4°C to 24°C room.

### 4.2. Antidepressant-induced acute analgesic effects on thermal hyperalgesia in ICS-exposed mice

Previous reports demonstrated that thermal hyperalgesia is elicited at P1 after ICS exposure and lasts for at least 12 days [13,14]. As shown in Figure 3, the nociceptive thermal threshold was significantly reduced and stable throughout experiments for 180 min. A single intrathecal injection of milnacipran (0.1 μg) had no effect on the nociceptive threshold in control mice (Figure 3A), but produced significant anti-hyperalgesic effects that persisted for at least 180 min post-injection at P1 (Figure 3A). This effect of milnacipran was dose-dependent in the range of 0.03-0.1 μg, but declined at 0.3 μg (Table 1). Statistical significance was observed at 0.1 and 0.3 μg. Similar results were observed with other antidepressants, such as amitriptyline (5-30 μg), mianserin (10 and 20 μg), and paroxetine (2-10 μg), as shown in Figures 3B-D and Table 1. However, with 20 μg of mianserin, a significant analgesic effect was observed at 60 min in the control mice, and anti-hyperalgesic effects were observed until 180 min (Figure 3C). Both amitriptyline and paroxetine showed significant anti-hyperalgesic effects, but no significance was observed at 180 min (Figures 3B, D).

### 4.3. Permanent relief of abnormal pain by repeated central administration

As the anti-hyperalgesic effect of milnacipran remained 180 min after intrathecal administration at day P1 after ICS stress (threshold: ~ 7.46 ± 0.2 s), we measured the nociceptive threshold at 11:00 a.m. on day P2. As seen in Figure 4, a significant anti-hyperalgesic effect still remained (threshold: ~ 7.67 ± 0.6 s). The second administration of milnacipran was performed at 11:30 a.m. The basal nociceptive threshold at 11:00 a.m. on day P3 further increased to 8.56 ± 0.8 s. The increase in basal threshold was maintained by daily administration of milnacipran. Complete recovery to the normal pain threshold was observed on P6, the day following the last administration, and lasted until P12. Similar complete reversals of hyperalgesia on P5 and P12 were observed after 5-day administrations of amitriptyline (15 μg), mianserin (20 μg), and paroxetine (5 μg), as seen in Table 2. Complete recovery was also observed with ICS-induced mechanical allodynia, even on P14, following a 5-day administration of the antidepressants (Figure 5).

### 4.4. Lack of beneficial effects by repeated systemic administration

When milnacipran was given by intravenous (i.v.) injection (10 mg/kg), there was a significant analgesic effect in the thermal nociception test at 30 min in control mice. However, there was no significant suppression in the ICS mouse model using this dose of antidepressant up to 180 min on P1 (Figure 6A). The absence of an ameliorative effect on ICS-induced hyperalgesia was also observed with amitriptyline (3 mg/kg, i.v.), mianserin (10 mg/kg, i.v.), and paroxetine (1 mg/kg, i.v.), despite producing significant acute analgesia at 30 min in control mice (Figures 6B-D). In addition, the repeated systemic administration of milnacipran for 5 days did not affect the basal threshold throughout the experiment (Figure 6E). Repeated administrations of amitriptyline, mianserin or paroxetine also did not provide relief from ICS-induced hyperalgesia (Table 3).

## 5. Discussion

Patients with FM exhibit widespread pain, with diverse symptoms, such as fatigue, depression, and sleep disturbance. Although the pathogenesis of FM is not clearly understood, certain biological stressors, such as autonomic nervous system disorder and psychological distress seem to be closely related to the development of FM [24]. An important role for such stressors is supported by studies using animal models in which rats or mice are subjected to stressors, such as chemical, sound, or surgery stress, which induce long-lasting abnormal pain [9-11,25]. Recently, we reported that ICS produces long-lasting thermal hyperalgesia and mechanical allodynia in mice [13,14]. The ICS-induced pain is bilateral and female-predominant (after gonadectomy) [13], which are also features found in FM patients [26].

In this study, mice subjected to ICS exhibited a transient increase in plasma corticosterone levels on P1. In contrast, there was no significant change in corticosterone levels in mice subjected to CCS. Considering that the abnormal pain in CCS mice was only transient, and not long-lasting [13,14], the rise in corticosterone levels in ICS mice likely played a role in the appearance of abnormal pain. A recent report suggests that the stress-induced increase in corticosterone concentration may be related to abnormal pain behavior in an FM-like animal model, possibly through a mechanism involving epinephrine release [27].

In our ICS model, the mice did not show significant changes in the tail-suspension test, a behavioral test designed to assess depression-like behavior [28]. This is in contrast to a study using less frequent temperature alternation (the SART model), in which mice exhibited hyperalgesia for only a week [29], and there was a transient reduction of immobility duration in forced swimming test, followed by gradual recovery in 5-6 days [30]. As the forced swimming causes a facilitation of immobility in an antidepressant-reversible manner [31], it is not clear whether the transient reduction of immobility duration reflects depression. From this point of view, the tail suspension test seems to be a better method for evaluation of depression-related despair behavior. Gabapentin and pregabalin are widely used to treat FM patients in the clinic [32,33]. These medicines alleviate abnormal pain and the accompanying fatigue and insomnia, without affecting depressive symptoms [33,34]. Therefore, the presence of depression-like behavior is unlikely to be necessary in animal models of FM. Consequently, the ICS model may be more clinically relevant than the SART model for evaluating long-term pain.

Various antidepressants have been used for FM in the clinic [35,36]. Recently milnacipran and duloxetine, serotonin/norepinephrine reuptake inhibitors, and serotonin-specific reuptake inhibitors have been approved for treating FM pain by the United States Food and Drug Administration. As the antinociceptive activities of these compounds are largely independent of their effects on mood, making them potentially efficacious for patients with or without depressive [37], it appears to reflect the importance of central descending monoaminergic pathways in pain regulation [38,39]. Recent studies revealed that polymorphisms in the 5-HT receptor, transporter, and metabolic enzyme can contribute to the etiology of FM [40-42]. The fMRI study also demonstrates that brain regions involved in descending pain inhibitory pathways appear to have decreased activity in FM patients [43]. Although serotonergic and/or noradrenergic pathways are well documented as descending pain inhibitory pathways [39], there is no report that the abnormality of such descending monoaminergic systems is observed in FM patients. However, it would be challenging to examine the effects of representative antidepressants on ICS-induced abnormal pain by introducing the drugs into the intrathecal space, very close to target regions.

Our study shows that the repeated intrathecal administration of different antidepressants gradually suppressed ICS-induced pain. The gradual reversal of abnormal pain may be related to the down-regulation of β-adrenoceptors or abnormal monoaminergic metabolism [44-46]. Alternative mechanisms may include the altered expression of multiple receptors and ion channels, such as the NMDA receptor, opioid receptors, and sodium channels [47-49]. It should be noted that the reversal of abnormal pain continued after the cessation of drug treatment, for each of the antidepressants tested. Although further investigation is required to clarify the molecular mechanisms of antidepressant action and to provide a permanent cure for ICS-induced abnormal pain, it is interesting to speculate that the chronic pain may be due to a vicious cycle of pain elicited by reduced inhibitory input from monoaminergic pathways. Thus, the rescue of pain-inhibitory mechanisms by repeated antidepressant treatment should halt chronic pain. Similar observations were made in our previous study using central administration of gabapentin [13,14]. In that study, using the ICS model, a single intracerebroventricular administration of gabapentin produced a 4-day period of anti-hyperalgesia. As the injection had no effect on peripheral nerve injury-induced neuropathic pain [13,14], and the gabapentin was unlikely to have remained in the brain for 4 days, it is interesting to speculate that the observed effect is due to the inhibition of the pain cycle, possibly through enhancement of inhibitory transmission. However, the present study demonstrates that systemic administration of various antidepressants had no significant beneficial effect on ICS-induced hyperalgesia, though they had a significant acute analgesic effect in control mice. As the clinically beneficial effects of oral antidepressants to FM patients were evident when they are treated for more than several weeks [50], the lack of effects of intravenous antidepressants in the present study may be attributed to the shortage of treatments (5 days). In this meaning it is surprising that only 5 days repetitive intrathecal treatments abolishes abnormal pain even after the cession of treatments. Furthermore, although the mechanisms underlying the lack of antihyperalgesic effect remain elusive, it may be worthwhile to investigate possible involvements of interference of spinal effects by peripheral pain facilitating serotonergic actions or by descending pain facilitating monoaminergic systems [39]. Thus, we expect that repetitive intrathecal administration of antidepressants are likely to be more effective at treating FM-like pain in mouse models.

Finally, this study demonstrates that the ICS model has similarities to clinical features of FM in terms of the sensitivity to analgesics or adjuvant analgesics. In our previous findings, we observed that the effective dose of gabapentin was 3 mg/kg for ICS-induced pain, but was over 30 mg/kg for nerve injury-induced neuropathic pain in mice [13,14], consistent with the fact that the clinically-effective dose of gabapentin for FM patients is lower than that for neuropathic pain [51]. In addition, we observed that ICS-induced thermal hyperalgesia was resistant to morphine treatment [13,14], consistent with the clinical evidence [52]. Considering that other experimental animal models of FM-like pain exhibit morphine analgesia (albeit with low potency) [53-56], the ICS model may be pharmacologically distinct from the others.

## 6. Conclusion

This study demonstrates that repeated intrathecal antidepressant treatment provides a complete cure of ICS-induced FM-like abnormal pain. Based on the pharmacological similarity of ICS-induced pain to clinical FM, the ICS model appears to be suitable for investigating the pathogenesis of FM and for evaluating therapeutic strategies for this debilitating illness.

## List of abbreviations used

aCSF: artificial cerebrospinal fluid; AUC: area under the curve: CCS: constant cold stress; FM: fibromyalgia; ICS: intermittent cold stress; PWL: paw withdrawal latency; PWT: paw withdrawal threshold.

## Competing interests

The authors declare that they have no competing interests.

## Authors' contributions

MN participated in the experimental designing, collection and analyses of data, and drafted the manuscript in equal contribution. HU and JN performed the statistical analyses and carried out surgical manipulation, data collection, and drafted the manuscript. KA and TM performed stress exposing and participated nociceptive behavior assay. SK measured plasma corticosterone levels. HU conceived of the study, participated in its design and coordination. All authors read and approved the final manuscript.
